# Pro-Moieties of Antimicrobial Peptide Prodrugs

**DOI:** 10.3390/molecules20011210

**Published:** 2015-01-13

**Authors:** Eanna Forde, Marc Devocelle

**Affiliations:** 1 Department of Clinical Microbiology, Royal College of Surgeons in Ireland, Dublin 9, Ireland; 2 Centre for Synthesis and Chemical Biology, Department of Pharmaceutical and Medicinal Chemistry, Royal College of Surgeons in Ireland, 123 St. Stephen’s Green, Dublin 2, Ireland; E-Mail: marcdevocelle@rcsi.ie

**Keywords:** antimicrobial, peptides, PEG, prodrug, antibody, charge

## Abstract

Antimicrobial peptides (AMPs) are a promising class of antimicrobial agents that have been garnering increasing attention as resistance renders many conventional antibiotics ineffective. Extensive research has resulted in a large library of highly-active AMPs. However, several issues serve as an impediment to their clinical development, not least the issue of host toxicity. An approach that may allow otherwise cytotoxic AMPs to be used is to deliver them as a prodrug, targeting antimicrobial activity and limiting toxic effects on the host. The varied library of AMPs is complemented by a selection of different possible pro-moieties, each with their own characteristics. This review deals with the different pro-moieties that have been used with AMPs and discusses the merits of each.

## 1. Introduction

Antimicrobial peptides (AMPs) are short, amphipathic peptides that play a crucial role in the innate immune system of all multi-cellular organisms [1]. They are generally 12–50 amino acids in length with most having a net positive charge due to an excess of basic residues such as lysine and arginine. This net charge, in combination with a significant proportion (up to 30%) of hydrophobic residues, is the basis of their antimicrobial activity [1,2,3]. AMPs with a net negative charge have also been described such as SAAP, but for the purposes of this review the focus will remain on cationic AMPs [4,5]. AMPs initially interact with anionic phospholipid head-groups of the bacterial cell envelope in an electrostatic manner. This can lead to either membrane perturbations or translocation across the membrane and interaction with various intracellular targets [6,7,8]. Several models exist for membrane disruption depending on the characteristics of the AMP. One is the barrel-stave model, where AMPs aggregate in a barrel-like ring in the bacterial membrane, forming an aqueous pore. Alternatively, in the toroidal pore model, AMPs induce a fold in the membrane. Finally, the carpet model involves a detergent-like effect induced by high concentrations of AMPs accumulated at the membrane surface [9].

While cell membranes have a similar function in bacteria, fungi, and mammals, their composition differs, a characteristic that is exploited by AMPs. AMPs show selectivity for association with membranes with large amounts of anionic lipids, such as those in bacteria, over other membranes enriched in cholesterol, such as those in mammals [10]. For example, 36% of the lipid content of *Staphylococcus aureus* membranes is represented by the anionic lipid phosphatidylglycerol, while on the other hand cholesterol makes up 45% of that of mammalian cells. Bacteria are completely lacking in membrane sterols such as cholesterol or the ergosterol found in fungal membranes. Cholesterol condenses the lipid bilayer and renders the adsorption of bulky amino acids such as tryptophan, found commonly in AMPs, energetically unfavourable [11]. The importance of this in the context of AMPs is demonstrated in one study where the binding of AMPs modified with multiple tryptophan residues was found to permeabilise liposomes mimicking bacterial membranes more so than those enriched in cholesterol, mimicking mammalian membranes [12].

The multiple modes of action of AMPs, including membrane depolarisation, pore formation, induction of degradative enzymes and disruption of intracellular targets, mean that bacteria may have a lower propensity for the development of resistance to AMPs compared to conventional antibiotics. This would potentially increase the lifetime of any exogenous AMP in clinical use and is one of the major rationales for their development as anti-infective therapeutics [13]. The issue of resistance to current therapies has led to increasing focus on the use of AMPs [3].

AMPs also have function beyond antimicrobial activity, a fact that is being increasingly recognised. For example it can be complemented by a chemotactic activity for phagocytes, and memory and effector T cells. AMPs can mediate the recruitment of immature dendritic cells, by direct chemotactic activity or by upregulation of chemokine production in macrophages, and promote maturation of these dendritic cells directly or indirectly by inducing production of inflammatory cytokines (IL-1β, TNFα) [14]. Although the latter activity results in the local release of pro-inflammatory cytokines, AMPs can also reduce the systemic production of TNFα, IL-1β and IL-6, and their global actions are generally consistent with a limitation of the inflammatory response and an enhancement of the innate and adaptive immune responses against microbial invasion [15,16]. For example, the human AMP LL-37 can bind to LPS at the site of infection, preventing it from interacting with TLR4 and having its pro-inflammatory effect [17]. While immunomodulatory effects are often seen with endogenous peptides such as LL-37 and hBD-3, they are not limited to them. The rationally-designed Innate Defense Regulator (IDR) peptides are able to induce chemokine release from peripheral blood mononuclear cells, demonstrate anti-inflammatory effects, and are protective against infection though possessing little direct antimicrobial activity themselves. The basis of these effects is less well understood than AMP antimicrobial activity but may be related to their ability to interact with cell membranes, translocate into cells and interact with intracellular targets. Direct receptor interaction may also play a role, such as with LL-37 and the formyl peptide receptor like 1 (FPRL-1) receptor [18].

There are a wide range of AMPs described in the literature. The main mammalian families include the defensins, cathelicidins, and histatins. AMPs are generally grouped by their structural characteristics, for example, defensins consist of parallel β-sheets linked by disulphide bonds and cathelicidins have a highly conserved precursor domain (but different final structures, including α-helices or disulphide bonded regions) [19]. The diversity of sequences is enormous; one species can potentially possess dozens of different AMPs, with no similar sequences found in related animals. This may be due to rapid evolution of sequences, with modification occurring as each species faces very different microbial challenges [1]. The result of this rapid evolution is a rich library of potential sequences, of varying characteristics and activities that may be harnessed for exogenous use against infection. However, despite the wealth in number and characteristics, the development of AMPs as antimicrobial agents has been hampered so far by a series of shortcomings. The main issues, apart from low bioavailability, include the cost of production, lability to proteases and unknown toxicology. For example, host AMPs such as LL-37 and hBD-3 are long sequences, 37 and 45 amino acids respectively, that would be expensive to produce commercially, and both are degraded by host proteases in conditions where their use would be required e.g., in cystic fibrosis (CF) [20,21]. Shorter peptide sequences will address some of the cost issues and approaches such as non-natural D-amino acids or PEGylation can prevent protease degradation. Toxicology can only be dealt with via careful selection of sequence and proper screening [10].

Significant research has been undertaken to develop AMPs as viable therapeutics for a host of infectious diseases. Several clinical trials have investigated the use of AMPs but no studies involving the systemic route have progressed further than early-phase. The therapeutic indices of AMPs can often be very narrow and most clinical trials have focused on topical applications. Some of the reasons for this include rapid metabolism and a lack of affinity for their targets [13,22,23,24]. To compound this, increased activity, e.g., due to increased hydrophobicity, is often at the expense of selectivity between eukaryotic and prokaryotic membranes, resulting in host toxicity [19]. For example, the β-hairpin peptides such as polyphemusin I, derived from the horseshoe crab, are extremely active with a Minimum Inhibitory Concentration (MIC) against *Pseudomonas aeruginosa* of 0.25 μg/mL. However, like many AMPs, they exhibit haemolytic activity at higher concentrations [25] and even endogenous AMPs such as LL-37 can be cytotoxic at high concentrations [26].

**Figure 1:**
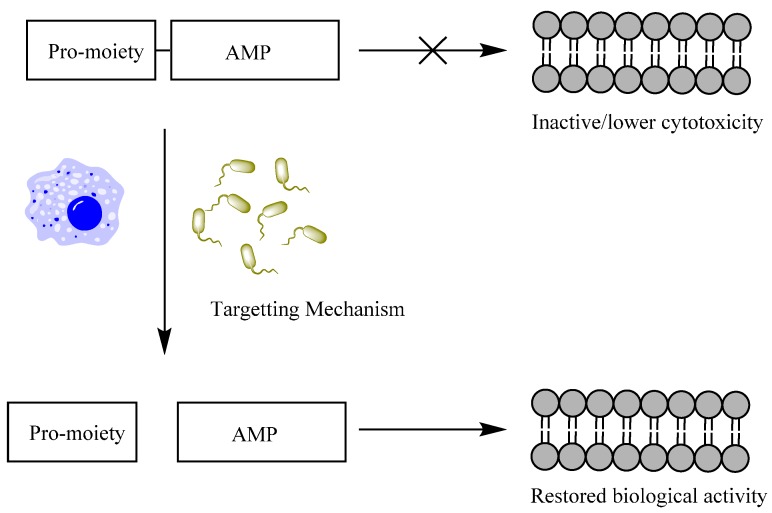
Overview of AMP prodrug model. The pro-moiety prevents the active AMP from interacting with cell membranes, both bacterial and mammalian, reducing both activity and cytotoxicity. The targeting mechanism, e.g., bacterial or host enzymes, releases the active AMP, restoring activity and limiting cytotoxic effects distal from the site of activation.

An alternative approach which may limit AMP toxicity and increase specificity would be to develop an AMP prodrug. Prodrugs are chemically-modified derivatives of active agents that are transformed *in vivo* to produce the active drug [27]. This would allow the activity of the AMP to be targeted to a specific bacterium or biological system and reduce cytotoxicity distal from the site of activation. This approach has parallels in nature. LL-37 is held in specific granules of neutrophils as the inactive hCAP-18. This is cleaved outside the cell to active LL-37 by the enzyme proteinase 3, released from azurophilic granules in neutrophils in response to infection. In this manner, the activity, and potential cytotoxicity, of the endogenous AMP can be limited until required to fight infection [28]. Applying this approach to exogenous AMPs would limit activity to the site of activation and reduce host cytotoxicity (see Figure 1). A number of pro-AMPs have been developed using a variety of approaches and several options exist for pro-moieties, including polyethylene glycol (PEG), oligoglutamic acid and small molecules. This review will discuss these pro-moieties, how the pro-AMP systems have performed, and the relative advantages and disadvantages of each modification.

## 2. Antimicrobial Peptide Prodrugs

The synthesis of AMP prodrugs can be achieved by a variety of means, but it essentially follows one of two approaches: activity may be modulated by manipulating the primary amphipathic character of the peptides via reduction of the net charge and/or the overall hydrophobicity. In some cases both approaches may be taken with one modification e.g., reducing the net charge by the addition of negatively charged pro-moieties will also reduce the global hydrophobicity. Many of the prodrug strategies detailed in the review will incorporate both approaches.

### 2.1. Net Charge Reduction

As stated above, antimicrobial peptides exploit an important difference between bacterial and mammalian cell membranes. Bacterial outer membranes have a net negative charge and higher transmembrane potential gradient (generally −140 mV at neutral pH) than those of mammals (−60 to −80 mV) [1,25,29]. Bacterial membranes are essentially populated with lipids containing negatively charged phospholipid headgroups. On the other hand, mammalian membranes are equally composed of positively and negatively charged lipids and have therefore no net charge. The different electrostatic interactions between AMPs and these membranes grants them a degree of selectivity [3] and allows them to disrupt bacterial cytoplasmic membranes, both Gram-positive and -negative [2]. In cell membrane-mimicking liposomes, the increased net negative charge of lipid membranes has been shown to increase AMP adsorption and AMP-induced leaking of liposomal contents compared to zwitterionic membranes, demonstrating the higher affinity of the AMPs for anionic lipid components [11]. The importance of this interaction is underlined by the fact that nearly all AMPs have a net charge in the range of +2 to +9 [16]. As a result of this, one prodrug approach has been the reduction of AMP net charge.

Peptides derive their net positive charge from their *N*-terminus amine and basic residues such as arginine and lysine. The importance of net charge to activity is underlined by colistin and its prodrug. This non-ribosomal peptide of *Bacillus polymyxa* is used against *P. aeruginosa* infections in cystic fibrosis. Its use was limited for many years over fears of nephrotoxicity but was reconsidered and re-employed with the emergence of bacterial resistance [30]. Its activity is dependent on its amphipathicity, and its mechanism of action is similar that of AMPs, involving electrostatic interaction with lipopolysaccharide (LPS) in the outer Gram-negative membrane. Its charge is derived from its diaminobutyric acid residues [31]. Currently it is delivered as its prodrug colistimethate via inhalation [32,33]. Colistimethate is inactive and less toxic due to the addition of sulfomethyl groups to the five primary amines of colistin, formed by reacting it with formaldehyde and sodium bisulfite [30]. This changes the net charge from +5 to −5, reducing both antimicrobial activity and host toxicity [34]. The sulfomethyl groups are hydrolysed *in vivo*, releasing active colistin and restoring the net positive charge [35]. However, sulfomethyl is not ideal as a pro-moiety. The release of the active colistin relies on spontaneous hydrolysis, an inefficient and non-selective process, releasing partially sulfomethylated derivatives alongside free colistin, with the net charges of the derivatives ranging from −3 (four attached sulfomethyl groups) to +3 (one attached group). In addition, factors such as pH and temperature have a major effect on release. For example, an *in vitro* study reported that only 31.2% of colistimethate was hydrolysed to colistin after 4 h in aqueous solution at 37 °C [30]. Commercially-available preparations of colistimethate may also differ between suppliers, with spontaneous hydrolysis resulting in undefined mixtures of substituted compounds with different activities [36].

Positive net charge as a basis of activity is not merely the domain of AMPs. In a similar manner, cell penetrating peptides (CPPs) rely on high net positive charge to translocate membranes and deliver covalently-attached conjugates such as fluorescent dyes, and can illustrate how AMPs may be modified. Indeed the reduction of the net positive charge of CPPs via the addition of anionic glutamic or aspartic acid residues has been shown to inhibit their cellular uptake. In one study, the insertion of a matrix metalloprotease (MMP)-targeted PLGLAG linker in between the CPP and anionic groups allowed MMP-2 and -9-dependent increases in CPP uptake to occur, the enzyme cleaving the anionic group and restoring the net positive charge [37].

A similar approach to that of the CPPs has been successfully employed with AMPs using the relatively facile addition of an oligoglutamic acid pro-moiety to reduce the net charge of the AMP P18 from +8 to +3, reducing its antimicrobial activity against *P. aeruginosa* and *S. aureus*, and lowering it haemolytic activity. Included in the design was a tri-alanine linker designed for cleavage by the CF-associated enzyme neutrophil elastase (NE), allowing NE-dependent restoration of antimicrobial activity. In order to prevent the cleavage of the active sequence by NE, P18 was synthesised from d-amino acids, leaving the linker and pro-moiety as enzyme-labile L-amino acids [38]. This design has been modified with an AAAG linker and extended to a series of other AMPs including HB43 and Bac8c, similarly demonstrating NE-reversible reductions in antimicrobial activity against *P. aeruginosa*, with both purified enzyme and that contained in CF bronchoalveolar lavage fluid. In addition, further cytotoxicity studies agreed with the previous haemolytic results, that the addition of the pro-moiety reduced host toxic effects [39].

The combination of oligoglutamic acid and an enzyme-targeted linker sequence can potentially be applied to a series of disease models, with the potential to target both host and bacterial enzymes for cleavage and active AMP release. As there has been some interest in using some AMPs as anticancer agents, the use of an anionic pro-moiety and an enzyme-targeted linker has also been employed to improve cancer cell specificity. MMP-2, an enzyme overexpressed in cancer cells, has been targeted with a peptide linker consisting of the sequence GPLGIAGQ. The pro-moiety, based on a sequence from magainin, DAEAVGPEAADEEKDED, reduced the net charge of the AMP buforin by eight units from +7 to −1. *In vitro* tests showed cleavage with MMP, and MMP-producing cancer cells were found to be susceptible to the pro-peptide. In contrast, cells that do not produce the enzyme demonstrated no toxicity in the presence of the prodrug [40]. Similarly, the amoebapore lytic peptide H-3 has been modified with c-terminal γ-linked glutamic acids, preventing the peptide from inserting into mammalian cell membranes and exerting an anti-cancer lytic effect. The prodrug is targeted at the carboxypeptidase prostate-specific membrane antigen (PSMA), which can remove terminally-linked γ-glutamic acids and is found in malignant prostate tissue. Anticancer activity was demonstrated in cells overexpressing PSMA, with little lytic effects on PSMA-negative cell lines [41]. *In vivo* studies using a mouse tumour xenograft model demonstrated reduced tumour size in treated mice compared to control and, in a separate experiment, no significant toxicity was observed in healthy mice after IV administration of a 30 mg/kg dose [42].

The major advantage of this approach is that, even with the incorporation of an enzyme-targeted linker, the final peptide remains relatively short. This is important when the cost of production is taken into account but also in terms of the ease in which the sequence can be produced and modified in a research setting. Solid phase synthesis and RP-HPLC can be used to produce highly-pure well-characterised final products, allowing for the generation of *in vitro* data quickly. One can then return and optimise the pro-AMP based on the *in vitro* results.

### 2.2. PEGylation

**Figure 2:**
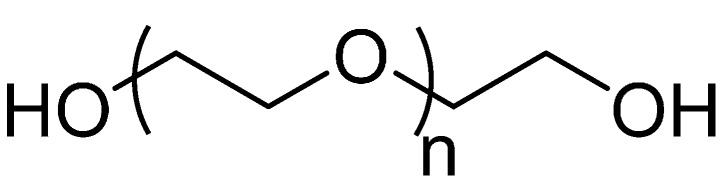
Poly(ethylene glycol) structure.

Poly(Ethylene Glycol) (PEG) is one of the most widely used polymers for improving the pharmacokinetic and pharmacodynamic properties of low molecular weight drugs, peptides, proteins and oligonucleotides (see Figure 2). It is synthesized via the nucleophilic attack of a hydroxide ion on the epoxide ring of ethylene oxide, leading to anionic polymerization. It can be covalently attached without crosslinking to drugs with a reactive functional group such as –COOH, –SH, –OH, or NH_2_ [43], making the attachment to a resin-bound, protected AMP relatively straightforward. PEG is currently used as a conjugate for many protein drugs, conferring increased aqueous solubility, reduced immunogenicity, prolonged half-life and higher specificity than the active agent alone [44]. Reversible PEGylation can be used to incorporate site-specific delivery such as controlled degradation in the target cell or tissue. This approach has been taken with many cytotoxic drugs, such as methotrexate, cisplatin, and paclitaxel [43,44]. PEG exerts its effects through a variety of mechanisms. One simple mechanism lies in PEG’s hydrophilicity; when bound to water, the attached PEG has a hydrodynamic radius 5–10 times that of a globular protein of similar molecular mass. The larger molecule is filtered at a slower rate in the kidneys, reducing renal clearance. The water-associated PEG also protects the attached active constituent from enzymatic degradation, protein interaction and fluctuations of temperature and pH. PEGylation can also prevent the attached drug from interacting with its target, but improvements in half-life can offset the effect of reduced activity [45]. For example, in anticancer therapy PEGylation will increase circulation time, and while activity is reduced, so are toxic effects. When this is used in conjunction with a targeting strategy it can improve the therapeutic index [43]. Many PEGylated anticancer therapeutics exploit the increased permeability of tumour vasculature and lack of lymphatic drainage to increase local drug concentrations, passively targeting tumours using a phenomenon called the enhanced permeability and retention (EPR) effect [44,46]. Once localized in the tumour, a release mechanism must then be employed for polymeric prodrugs based on PEG or other polymers such as *N*-(2-hydroxypropyl) methacrylamide HMPA e.g., exploiting lysosomal enzymes such as Cathepsin B with a peptide linker of sequence GFLG [44,47,48], or lower pH levels of tumour cells with a hydrazone linker [49].

PEG may be easily incorporated into resin-bound peptides, using supra-stoichiometric concentrations of the desired-length PEG in conjunction with solid support, allowing the diffusion of the polymer in its matrix. However, while widespread in anticancer therapies, PEG has not been used as extensively with AMPs. As with other molecules, PEGylation can decrease the binding of peptides to their biological targets, cell membranes in the case of AMPs, reducing antimicrobial activity but also cytotoxicity and haemolysis [50]. This reduction in both host- and bacterial-targeting effects has been exploited to improve the selectivity of AMPs. While not incorporating a prodrug release mechanism, a conjugate of the AMP magainin and 5 kDa PEG had far lower cytotoxicity against CHO-K1 cells at 100 μM but only a four-fold reduction in MIC against *Escherichia coli* and *Staphylococcus epidermidis* [51]. A conjugate of tachyplesin and 5 kDa PEG displayed similar reductions, again with no release mechanism incorporated into the design. However, while reducing the cytotoxicity of tachyplesin, PEG had a major deleterious effect on antimicrobial activity, reducing the MIC against *E. coli* from 0.5 μg/mL to 32 μg/mL [50]. Reductions in haemolysis compared to control were noted in a PEGylated AMP, KYE28, and was inversely related to length of the attached PEG. Improved selectivity was demonstrated in a blood mixture supplemented with a bacterial inoculum, where PEGylation reduced haemolysis while maintaining antimicrobial effect. This ran in parallel with reductions seen in adsorption to model lipid membranes (both anionic and zwitterionic) and antimicrobial effect with increasing PEG length. Also noted was a modest reduction in LPS binding with PEGylation, which was interesting from an immunomodulatory aspect. Furthermore, while the parent peptide reduced LPS-induced NF-κB activation of macrophages, the PEGylated peptide was moderately less effective, indicating that the pro-moiety was interfering with immunomodulatory effects [52]. Using a short PEG, another group was able to demonstrate modest reductions in cytotoxicity with small decreases in antimicrobial activity. They proposed that PEGylation was able to reduce the association with zwitterionic membranes, such as those in mammals, while leaving the interaction with anionic bacterial membranes relatively unaltered. They demonstrated that the PEGylated AMP, compared to the unaltered AMP, had an increased preference for binding with anionic model membranes than their zwitterionic counterparts. Interestingly, they demonstrated that the PEG used was unable to prevent AMP degradation by rat lung proteases and that the PEG prodrug was in fact more labile than the free AMP, proposing that in this case PEGylation may interfere with the AMP’s ability to self-associate and reduce its stability [53]. Conversely, the use of short PEGs has been shown to protect against degradation by chymotrypsin and serum proteases, which is important when one considers the impediment to AMP development that protease-lability represents. While the modified peptide showed a reduction in antimicrobial activity compared to the unconjugated AMP, it maintained antimicrobial activity better in serum, which had a major deleterious effect on the activity of the parent AMP. The authors of the study note that the PEG length is below that which is usually required to extend peptide half-life (2 kDa and higher) but that the short PEG may still shield the peptide core from degradation [54]. The ability of PEG to protect from proteolytic degradation may depend on the enzyme in question and the length of PEG used. Likely degradative enzymes will have to be taken into account when considering the length of PEG for use in the prodrug system.

While PEGylation alone may be used to improve the selectivity of AMPs, the inclusion of targeted release mechanisms can take advantage of the reductions in cytotoxicity while circumventing the issue of reduced antimicrobial activity. The covalent linkage of PEG or other polymers to the AMP can be designed for cleavage under certain physiological and enzymatic conditions to ensure controlled release of the active peptide and limitation of host toxic effects, producing a prodrug. As mentioned above, this can take the form of a covalent bond such as a pH-labile hydrazone [49], or a short amino acid sequence targeted at an enzyme. Nollman *et al.* produced a series of PEG-AMP conjugates targeted for release by trypsin-like proteases found in serum using a GARSG linker sequence. The active sequences had previously been optimised for stability against mouse serum proteases. This provides a controlled systemic release of active AMP. It was again found that PEGylation will reduce antimicrobial effects, with MICs increasing from 0.8 μM to 9.3 μM with the conjugation of a 5 kDa PEG and a linker. The addition of mouse serum containing the target proteases was able to restore activity and reduce the MIC back to 0.6 μM. Release kinetics varied between pro-AMPs and depended on PEG length and linker, with the AMP Api301 attached via a GSRG linker sequence being released at a faster rate from a 750 Da PEG than a 5 kDa analogue, consistent with lower protein binding seen elsewhere. Longer linker sequences also slightly retarded the rate of release [55].

Interestingly, the Therapeutic Index increase associated with the PEGylation of AMPs might not be achieved with some candidates. For example, conjugation of a 2 kDa PEG to P18 through an AAAG linker targeted at neutrophil elastase increased the MIC against *P. aeruginosa* from 2 μg/mL to >128 μg/mL, agreeing with many of the studies detailed above. However, the susceptibility of the linker to NE cleavage was much reduced compared to an oligoglutamic acid prodrug system, with no cleavage observed after 1 h incubation with 50 μg/mL NE. Compounding this, there was no observed reduction in cytotoxicity against CFBE41o- cells (CF host epithelial cells) with an IC_50_ of 38.7 μM, compared to 35.5 μM for the active cleavage product AAG-P18, where previously oligoglutamic acid had been observed to reduce toxicity against the same cell line [39]. Hence, the PEG was able to prevent the release of the active AMP while not reducing its cytotoxic effects [56]. While PEGylation did not produce a successful prodrug in that instance, the results of previous studies indicate that it may have a useful role in future AMP prodrugs, warranting further study with a suitable enzyme-linker system and an optimised PEG and linker length. Like oligoglutamic acid, it can be produced using solid-phase synthesis, allowing highly-pure products to be obtained, although a polymeric prodrug would likely be relatively large, fitting the description of a nanomedicine, according to the definition of the European Science Foundation [43].

### 2.3. Antibody and Pheromone Conjugates

In certain clinical scenarios it may be highly desirable to target the activity of an AMP at a pathogenic bacterium with minimal effect on normal commensal flora, such as in the mouth or the colon. Several strategies have been employed to achieve this. While they are not based on bioreversible derivatives, they may be taken under consideration in future AMP prodrug models. The 28 residue AMP SMAP was linked to immunoglobulin G antibodies specific for the outer surface of the bacterium *Porphyromonas gingivalis*. While the authors of the study were unable to determine the number of AMPs linked to the antibody, they were able to demonstrate preferential killing against *P. gingivalis* in a simulated microbial community at 20 μg/mL. Specificity was lost, however, at the higher concentration of 50 μg/mL [57]. A consideration for antibody conjugates is the improvements in activity relative to the increase in product size and cost of production, with the final therapeutic likely to have a molecular mass in the region of 150 kDa. While there is scope to incorporate this into a prodrug model, this approach does also not allow for synthesis by solid-phase chemistry, and requires recombinant strategies that may prove difficult to design and implement. For example, the AMP dhvar5 was conjugated to a llama heavy-chain antibody against *Streptococcus mutans* with a linker sequence targeted at the endoproteinase factor Xa. While it was demonstrated that the linker allowed factor Xa-dependent restoration of antimicrobial activity using a short peptide pro-moiety, purification and testing of the larger antibody conjugate was not possible due to low yield, product degradation, and poor bacterial vector growth [58].

In a similar manner to antibody conjugates but using a smaller targeting moiety, antimicrobial peptides, both AMPs [59] and longer, pore-forming bacteriocins [60,61] have been conjugated to bacterium-specific pheromones. It has been demonstrated that, by linking short pheromones to an AMP, one can target two specific bacteria. In one study the targeting moieties M8 (TFFRFLNR) and KH (KKHRKHRKHRKH) were attached to the AMP BD2.20 with tri-glycine linkers. This allowed selective killing of both *P. aeruginosa* and *S. mutans* in a mixed culture, leaving other bacteria relatively untouched [62]. These short pheromones are promising as pro-moieties as they can be easily incorporated into the synthesis of AMPs and as seen in the previous study, can reduce antimicrobial activity against non-target bacteria. The tri-glycine linker could also potentially be converted into an enzyme-labile sequence to allow cleavage of the targeting pro-moiety, perhaps by a target bacterial enzyme to enhance specificity by releasing the more active AMP once the prodrug has reached the target bacterium. This pheromone combination may allow the targeting of more ubiquitous bacterial enzymes that would otherwise be too commonplace to allow selective killing. Again, this modification would result in a relatively short pro-AMP, making it an attractive prospect.

### 2.4. Antibiotic Conjugates

As an alternative to inert PEG or oligoglutamic acid, the prodrug can incorporate an active antibiotic as a pro-moiety. The conjugation can use characteristics of both components to overcome issues of toxicity and selectivity. For example, vancomycin has been combined with the AMP magainin using click chemistry. By targeting Lipid II, the antibiotic has a much higher affinity for Gram-positive membranes compared to the non-selective electrostatic attraction of magainin. Magainin, like all AMPs, can take advantage of the lower propensity for resistance to develop in bacteria. Combining the characteristics of both active agents, the conjugate had an MIC of 16 μg/mL against vancomycin-resistant *Enterococci* compared to 128 μg/mL for vancomycin alone. While illustrating some of the benefits of antibiotic conjugation, this design does not incorporate a release mechanism for the AMP and doesn’t transiently inactivate the 2 agents. There is scope for further modification and, as with bacterial pheromones, vancomycin may be taken into consideration for future co-drug models [27,63]. However, activity would potentially be limited to Lipid II-containing Gram-positive bacteria.

Additional design characteristics can incorporate the antibiotic as a targeted pro-moiety. Conjugating antibiotics can provide a reduction in AMP net charge by masking the N-terminus amine and via the negative charge of functional groups such as –COOH, inactivating the AMP in much the same way as oligoglutamic acid. The antibiotic can be used in a manner that doesn’t address its normal target but rather act solely as a pro-moiety, ensuring the release of the intact active AMP to kill the bacterial target. One such conjugate involved the AMP Bac8c and the antibiotic cephalothin, which reduces the net cationic charge from +4 to +2, the prodrug having lower antimicrobial activity than both separate constituents in the absence of the target enzyme, β-lactamase. This prodrug can be targeted at β-lactamase-producing bacteria such as β-lactam resistant *E. coli*, where the enzyme will cleave the β-lactam bond of the antibiotic and release the active AMP. This resulted in an inactivated cephalothin component, precluding synergy, but directed the peptide activity against β-lactamase producing bacteria. Cleavage by the enzyme has been demonstrated with purified β-lactamase, but the prodrug showed reduced activity compared to Bac8c alone, with MIC values against *E. coli* of 12 μM *versus* 2.1 μM respectively. However, the activity was further reduced in a control with a stable oxime linker, uncleavable by β-lactamase (MIC > 24.8 μM), demonstrating a degree of enzyme-dependent activity. As the intact prodrug was still moderately active, further modification of the pro-moiety, potentially through the introduction of an additional negative charge, is required. This would ensure that the intact prodrug does not contribute towards the activity [64].

This approach also has scope for the inclusion of an enzyme-labile linker into the design. As above, the conjugation of antibiotics to the N-terminus amino acid may be achieved through click chemistry, allowing the possibility for the cleavage products to include both active AMP and antibiotic, ensuring that both work in synergy. The final conjugation step with the antibiotic may potentially be quite difficult and may result in reduced yields. This should be taken into account when this pro-moiety is being considered.

### 2.5. Other

In a manner similar to antibiotic conjugation, one can produce AMP prodrugs using an additional therapeutic agent as a pro-moiety, one that has an alternative target to infection and will separate from the AMP at the target site and provide dual action. For example, the non-steroidal anti-inflammatory drug (NSAID) 5-amino phenylacetic acid was conjugated to an analogue of the AMP temporin A. This was achieved using an azo bond targeted at the enzyme azoreductase of anaerobic bacteria such as *Clostridium difficile*, with a view of treating infection in the colon.

As with antibiotic conjugation, this lowers the net charge of the AMP by 2 units, with the N-terminus amine masked and a carboxyl present in the drug. This would limit the antibacterial activity of the peptide but also prevent the anti-inflammatory effects of the NSAID, each agent acting as a pro-moiety for the other in this co-drug until they are separated from each other in the colon. The design also aims to avoid many of the ulcerative side effects of the NSAID which, when released, will protect against pro-inflammatory effects of bacterial toxins. Biological activity, however, has not been determined [65].

**Figure 3:**
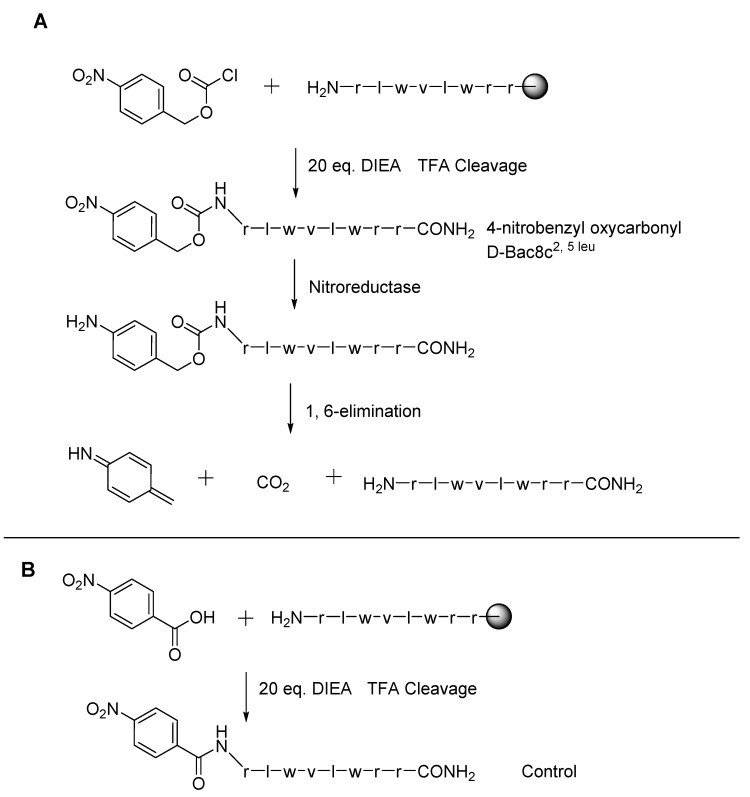
(**A**) Synthesis of 4-nitrobenzyl oxycarbonyl D-Bac8c^2,5 leu^ and release mechanism by nitroreductase of the active from the prodrug. (**B**) Synthesis of the control. Both syntheses are carried out by solid phase synthesis, the final products being isolated by cleavage from the resin with 80% trifluoroacetic acid and scavenging agents.

Nitroreductase enzymes in *C. difficile* could also be the target of AMP prodrugs. In a manner similar to the temporin A conjugate, D-Bac8c^2,5 leu^ was reacted by our group with *para*-nitrobenzyl chloroformate to produce 4-nitrobenzyl oxycarbonyl D-Bac8c^2,5 leu^ (see Figure 3). As above, this masks the *N*-terminus amine, reducing the net charge by one unit. The design aims to restore the active peptide upon contact with the bacterium of interest. With reduction by the enzyme of the aromatic nitro group to the corresponding aniline intermediate, the peptide is released after the intermediate spontaneously decomposes by 1,6-elimination. This was found to reduce the MIC against *C. difficile* from 2 μg/mL to 16 μg/mL. However, a similar reduction in activity was seen with an uncleavable control (produced by reacting the peptide with *para*-nitrobenzoic acid) (MIC of 16 μg/mL). This indicated a reduction in activity by the pro-moiety but no restoration of activity by the target enzyme in *C. difficile* (unpublished data).

Simple modifications such as this, and similarly for antibiotic conjugation, require the active AMP to have a relatively low net charge, as the reduction effected by the pro-moiety is modest. While Bac8c is a good candidate for this, the number of available active sequences is limited for this approach. An alternative application may be with lysine-containing AMPs, where the nitrobenzylcarbamate pro-moiety can mask the ε-amino groups and reduce the number of positive charges accordingly.

## 3. Conclusions

**Figure 4:**
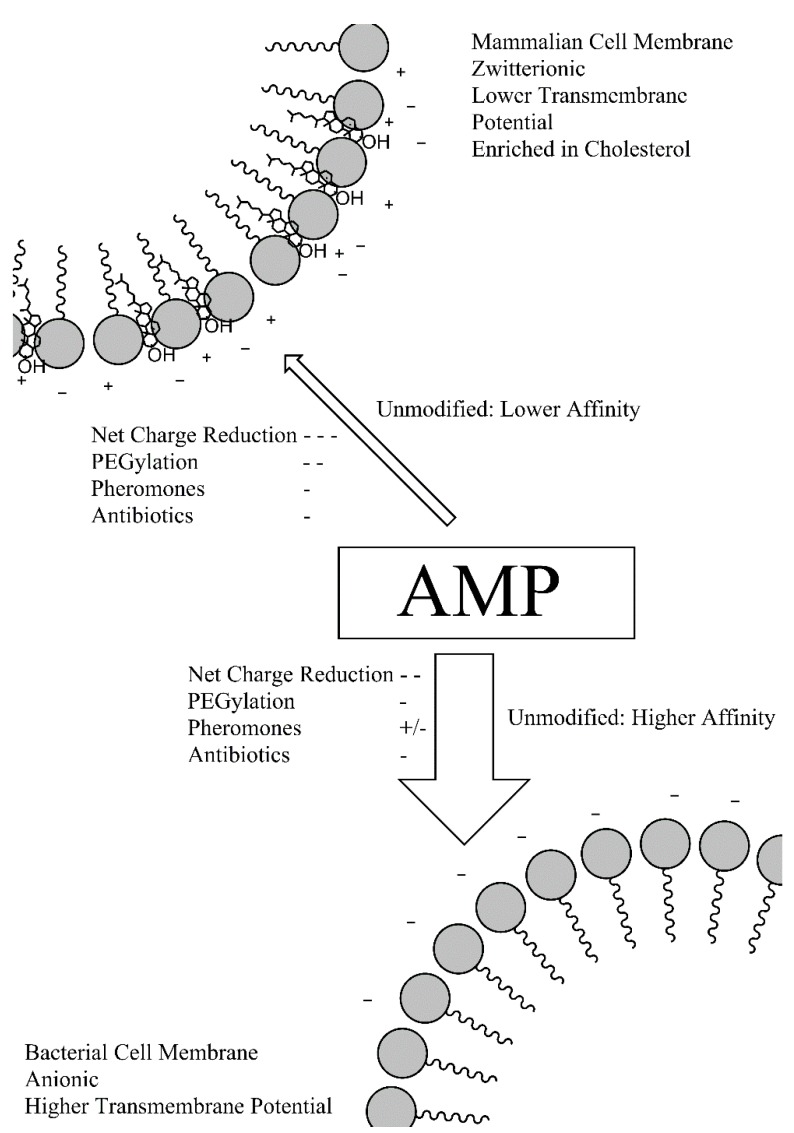
Overview of the effects of the different pro-moieties on the affinity of AMPs for mammalian and bacterial cell membranes.

The prodrug approach has the potential to greatly expand the number of AMPs suitable for anti-infective therapy. Highly-active AMPs that would otherwise be too cytotoxic under normal circumstances may be reconsidered for use as a prodrug that targets activity to a particular biological system. Several options for pro-moieties, with varying characteristics, have been investigated. Figure 4 illustrates how these different conjugates may be used to decrease the absolute affinity of AMPs for cell membranes, reducing host cytotoxicity and, in many cases, antibacterial activity. While many of the modification such as PEGylation will increase the selectivity of AMPs by increasing the relative affinity for bacterial cell membranes, the accompanying loss of absolute bactericidal activity necessitates the incorporation of a release mechanism for the active AMP *i.e*., the synthesis of AMP prodrugs. The various enzymatic targets used by the different prodrug models underlines how AMP activity can be tailored to specific bacterium or region of the body, limiting cytotoxic effects of the already selective AMPs. Considering that, along with potential cytotoxicity, economic considerations are a major barrier to the clinical AMP development, any pro-moiety will ideally not lengthen the AMP to a great extent and increase the cost of production. The ease at which the prodrug can be synthesised should also be taken into account. The literature has demonstrated pro-AMP strategies incorporating both charge reduction and PEGylation can produce significant reductions in cytotoxicity while allowing targeted release. Both are relatively simple to incorporate into solid-phase synthetic strategies and produce products that are relatively short in comparison to some other conjugates. PEG and oligoglutamic acid also provide a greater masking of activity compared to some of the shorter pro-moieties such as antibiotic conjugates. Based on the available information in the literature, both of these pro-moieties may be considered the most attractive and represent a promising avenue for the development of AMPs as antibacterial therapeutics. Further optimisation of other pro-moieties such as antibiotic conjugates may also yield better AMP prodrugs.

Considering the complexity of the targeted biological systems, an important future milestone in the development of pro-AMPs would be an in-depth *in vivo* study of not just the antimicrobial activity of the pro-AMP but also the activation by the target biological system, including analysis of the released active peptide in biological samples such as BAL fluid or serum. The reduction in toxic effects, if any, afforded by the modification must also be investigated. In addition, something that has not yet been studied in detail is the potential effects of the prodrug approach on the other effects of AMPs *i.e*., immunomodulatory activity, which may be a potential source of host toxicity with exogenous application. When one considers that membrane association and translocation may play a role in the immunomodulatory mechanism, it is possible that the addition of a pro-moiety may reduce these effects. Furthermore, where the immunomodulatory effect is the result of binding to immunogenic molecules such as LPS, the possibility that the pro-moiety will reduce this effect must be investigated [52]. Another important design consideration is whether the active sequence will be resistant to degradation by both target and off-target enzymes. AMP prodrug systems may rely on high concentrations of host or bacterial enzyme for activation, such as NE in cystic fibrosis, which could have the potential to degrade the active sequence. Strategies to avoid this must be incorporated into design, whether it be via PEGylation [54], D-amino acids [38], a protease-resistant sequence [55], or otherwise. These approaches, particularly D-amino acids, may also increase the future cost of production of pro-AMPs and it must be carefully considered whether they are necessary.
